# Comprehensive genotypic, phenotypic, and biochemical characterization of GOT2 deficiency: A progressive neurodevelopmental disorder with epilepsy and abnormal movements

**DOI:** 10.1016/j.gim.2025.101587

**Published:** 2025-09-23

**Authors:** Hannah M. German, Maha S. Zaki, Muhammad A. Usmani, Irem Karagoz, Stephanie Efthymiou, Mohamed S. Abdel-Hamid, Haya Abdelhafez Arabiyat, Amama Ghaffar, Mohsin Shahzad, Hans van Bokhoven, Zubair M. Ahmed, Omid Yaghini, Neda Hosseini, Maede Majidinezhad, Shahryar Alavi, Marjolein Bosma, Melissa H. Broeks, Dilşad Türkdoğan, Mohnish Suri, Laiz Laura de Godoy, Nanda M. Verhoeven-Duif, Sheikh Riazuddin, Joseph G. Gleeson, Cesar Alves, Judith J.M. Jans, Saima Riazuddin, Henry Houlden, Reza Maroofian

**Affiliations:** 1 Section Metabolic Diagnostics, Department of Genetics, University Medical Centre Utrecht, Utrecht University, Utrecht, The Netherlands; 2 Clinical Genetics Department, Human Genetics and Genome Research Institute, National Research Centre, Cairo, Egypt; 3 Department of Otorhinolaryngology Head and Neck Surgery, School of Medicine, University of Maryland, Baltimore, MD; 4 Department of Biotechnology, Kohsar University Murree, Pakistan; 5 Department of Molecular Biology, Shaheed Zulfiqar Ali Bhutto Medical University, Islamabad, Pakistan; 6 Department of Neuromuscular Diseases, UCL Queen Square Institute of Neurology, University College London, London, United Kingdom; 7 Medical Molecular Genetics Department, Human Genetics and Genome Research Institute, National Research Centre, Cairo, Egypt; 8 Ministry of Health, Al-Hussein Salt New Hospital, As-Salt, Jordon; 9 Centre of Excellence in Molecular Biology, University of the Punjab, Lahore, Pakistan; 10 Department of Human Genetics, Donders Institute for Brain, Cognition and Behavior, Radboud University Medical Centre, Nijmegen, The Netherlands; 11 Department of Molecular Biology and Biochemistry, School of Medicine, University of Maryland, Baltimore, MD; 12 Child Growth and Development Research Center, Research Institute for Primordial Prevention of Non-Communicable Disease, Isfahan University of Medical Sciences, Isfahan, Iran; 13 Department of Pediatric Neurology, Isfahan University of Medical Sciences, Isfahan, Iran; 14 Palindrome, Isfahan, Iran; 15 Marmara University, Medical Faculty, Department of Pediatric Neurology, Istanbul, Türkiye; 16 Clinical Genetics Service, Nottingham University Hospitals NHS Trust, Nottingham, United Kingdom; 17 Department of Radiology, Perelman School of Medicine, University of Pennsylvania, Philadelphia, PA; 18 Jinnah Burn and Reconstructive Surgery Center, Allama Iqbal Medical College, University of Health Sciences, Lahore, Pakistan; 19 Department of Neurosciences, University of California, San Diego, La Jolla, CA; 20 Rady Children’s Institute for Genomic Medicine, San Diego, CA; 21 Department of Radiology, Boston Children’s Hospital, Harvard Medical School, Boston, MA

**Keywords:** Epilepsy, GOT2, Malate-aspartate shuttle, Mitochondrial disorders, Neurodevelopmental disorder

## Abstract

**Purpose:**

Glutamic-oxaloacetic transaminase (GOT), also known as aspartate aminotransferase, catalyzes the reversible transamination of oxaloacetate and glutamate to aspartate and α-ketoglutarate. Two isoforms, cytosolic (GOT1) and mitochondrial (GOT2), are integral to the malate-aspartate shuttle, a key regulator of intracellular redox homeostasis. Recently, 5 patients with biallelic variants in *GOT2* were described, presenting with developmental and epileptic encephalopathy.

**Methods:**

We report 11 additional patients with homozygous *GOT2* variants, along with additional data from 4 previously reported patients. Through genetic, clinical, and biochemical analyses, we further characterize the phenotypic spectrum of GOT2 deficiency.

**Results:**

Most patients exhibited progressive neurodevelopmental delay, severe to profound intellectual disability, infantile epilepsy, progressive microcephaly, and hypotonia evolving into spasticity with axial hypotonia. Dysmorphic features included narrow foreheads, broad nasal tips, and tall or pointed chins. Neuroimaging revealed 2 severity groups based on cerebral volume loss and myelination defects. Thinning of the corpus callosum and white matter abnormalities were common. Biochemical profiling identified low aspartate and high glycerol-3-phosphate in dried blood spots as potential screening markers. Patient fibroblast cells showed reduced serine and glycine biosynthesis, rescuable by pyruvate supplementation.

**Conclusion:**

These findings expand the phenotypic spectrum of GOT2 deficiency, establish it as a cause of developmental epileptic encephalopathy, and propose novel biomarkers for diagnosis and treatment.

## Introduction

Glutamic-oxaloacetic transaminase, also known as aspartate aminotransferase (GOT; EC 2.6.1.1), catalyzes the reversible transamination of oxaloacetate and glutamate to aspartate and α-ketoglutarate in a pyridoxal-5′-phosphate-dependent reaction. Two isoforms exist: a cytosolic form (GOT1) and a mitochondrial form (GOT2), both integral to the malate-aspartate shuttle (MAS), a key regulator of intracellular redox homeostasis.^1,2^ The MAS facilitates the net transfer of NADH across the mitochondrial membrane and recycling of cytosolic NAD^+^ , processes critical for glycolysis, de novo serine biosynthesis, oxidative phosphorylation, and ATP production.^3^ Additionally, GOT2 catalyzes the main intracellular source of aspartate, a precursor for purine and pyrimidine biosynthesis and an essential substrate for the urea cycle.^4,5^

Biallelic missense variants in *GOT2* (OMIM 618721) were first described in 2019 by van Karnebeek et al,^6^ in 4 pediatric patients from 3 unrelated families. These patients presented with early-onset developmental epileptic encephalopathy (DEE82; 618721), hypotonia, feeding difficulties, and global developmental delay, often predating seizure onset in the first year of life. Shared features included severe intellectual disability with absent speech, spastic tetraplegia, microcephaly, poor growth, and recurrent infections. Brain imaging consistently revealed cerebral atrophy, a thin corpus callosum, cerebellar hypoplasia, and white matter abnormalities. Laboratory findings showed elevated serum lactate, hyperammonemia, lactic acidosis, and low plasma serine in the most severely affected patient. Functional studies in patient-derived fibroblasts demonstrated impaired de novo serine biosynthesis, which was rescued by pyruvate supplementation in GOT2 knockout HEK293T cells, indicating a redox-dependent mechanism. Seizures were responsive to pyridoxine and L-serine supplementation.

More recently, Çapan et al^7^ reported a fifth patient, a 29-year-old male with compound heterozygous missense *GOT2* variants. This patient presented with DEE, autistic features, and profound intellectual disability, alongside high plasma lactate, hyperammonemia, and low asparagine and methionine levels. Although pyridoxine treatment was attempted, it was discontinued after 3 weeks because of adverse effects.

Here, we describe 11 additional patients with biallelic pathogenic *GOT2* variants from 6 new families, as well as extended clinical and biochemical data from 3 previously reported families. Our findings significantly expand the genotypic and phenotypic spectrum of GOT2 deficiency, identify novel metabolic biomarkers, and suggest potential therapeutic targets. Together, these insights aim to facilitate earlier diagnosis and improved management of this rare condition.

## Materials and Methods

### Phenotypic and genetic evaluation

**Figure 1:**
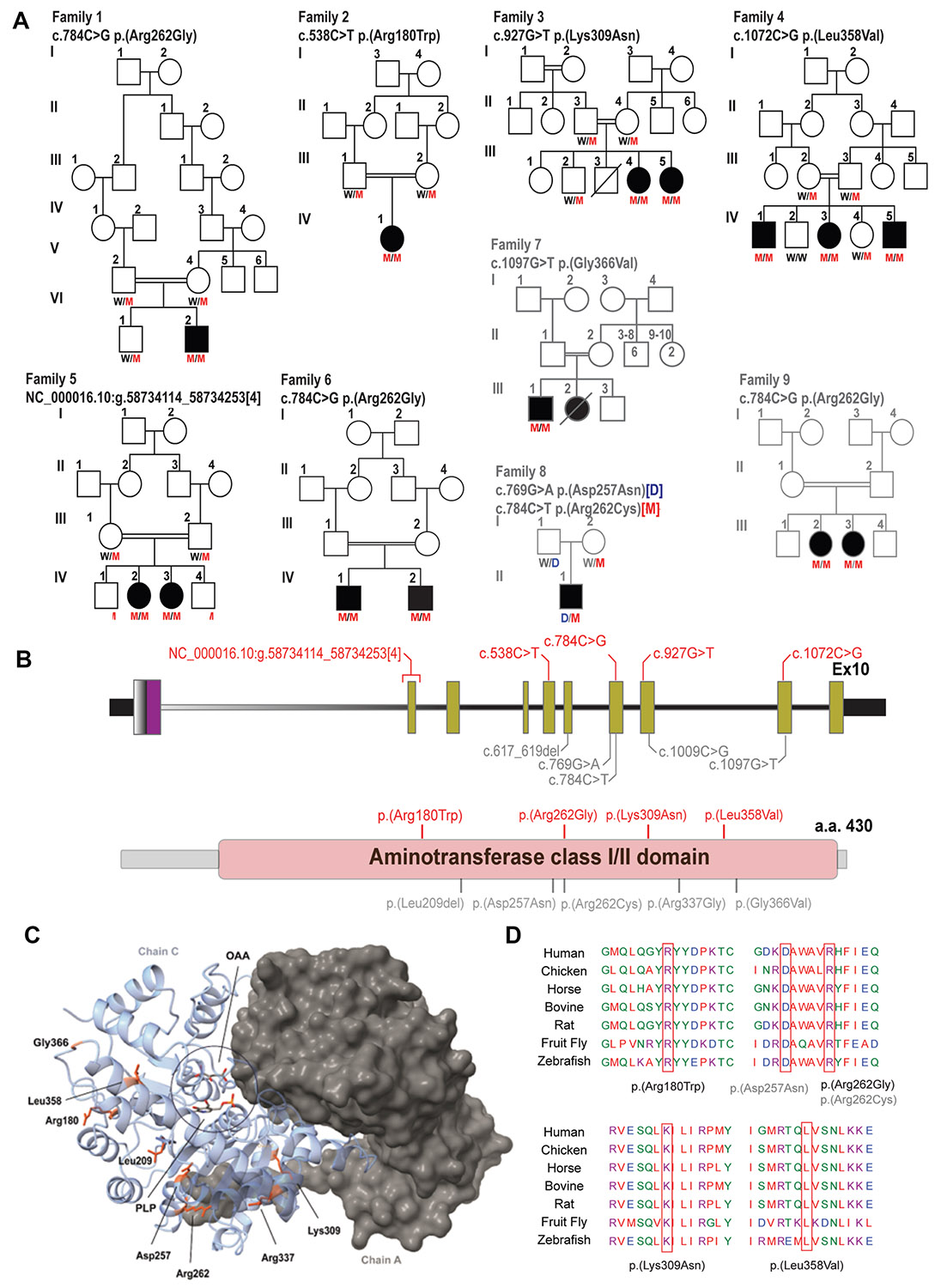
Genetic and structural overview of *GOT2* variants. A. Pedigrees of 6 newly identified families with *GOT2*-related DEE, as well as 3 previously reported families (shown in gray). B. Schematic depiction of *GOT2* DNA sequence and primary protein structure with locations of variants identified in this study (indicated in red) and in previous reports (indicated in gray; details listed in Table 1). C. Structural depiction of the reported missense variants within the GOT2 homodimer (chains A and C). Human GOT2 template structure was obtained from RSCB Protein Data Bank (PDB: 5AX8). Ligand coordinates for OAA were obtained from PDB: 3PDB. PLP ligand coordinates were obtained from PDB: 8SKR. Residues in which variants were reported in patients are marked in orange. OAA, oxaloacetate; PLP, pyridoxal 5′-phosphate. D. Cross-species evolutionary conservation of the GOT2 amino acids in which variants were identified and their surrounding sequences.

Comprehensive clinical, genetic, and biochemical data were collected from 11 affected individuals spanning 6 families, identified through GeneMatcher and collaborative data sharing worldwide (Figure 1A). Additionally, follow-up clinical and biochemical data were incorporated for 4 individuals across 3 previously reported families, indicated in gray in Figure 1A. Furthermore, we included published data from 1 additional previously reported patient, although no new information was available for this case.

The study was approved by the Institutional Review Boards of all participating institutions, with informed consent obtained from all families. A standardized proforma was used to collect detailed familial and medical histories. Affected individuals were assessed by physicians for anthropometric measurements, developmental milestones, and other neurological and physical features. Facial photos were reviewed by a dysmorphologist, and brain magnetic resonance imaging (MRI) scans were evaluated by a neuroradiologist and a pediatric neuroradiologist.

Genomic DNA was extracted from peripheral blood samples, and exome sequencing was performed at multiple participating research and diagnostic genetic laboratories utilizing the GRCh38 reference genome build obtained from UCSC Genome Browser.^8^ Bioinformatic analysis and variant curation followed a thorough multistep filtering process, as previously described, with segregation analysis conducted using Sanger sequencing.

### Cell culture

GOT2 patient and heterozygous fibroblasts were maintained in Dulbecco’s Modified Eagle Medium, high glucose, GlutaMAX, pyruvate (Thermo Fisher Scientific) supplemented with 10% v/v heat-inactivated fetal bovine serum (Thermo Fisher Scientific) and 1% v/v penicillin-streptomycin (10,000 U/ml; Thermo Fisher Scientific). Cells were kept in a humidified incubator at 5% CO_2_ and 37 °C and passaged upon reaching confluence. Medium was replaced every 48 hours.

### Western blot

GOT2 patient and heterozygous fibroblasts were harvested in triplicate in radioimmunoprecipitation buffer consisting of 0.05M Tris, 0.15 M NaCl, 1% NP-40, 0.5% sodium deoxycholate and 0.1% SDS, supplemented with protease inhibitor (1:200; Sigma-Aldrich), and 2 mM NaF phosphatase inhibitor. Samples were agitated for 30 minutes at 4 °C, after which they were centrifuged for 10 minutes at 14,000 rpm. The supernatant was transferred to a fresh Eppendorf tube and stored at −80 °C. For western blot, samples were diluted to the lowest protein concentration as determined by a bicinchoninic acid protein assay (Thermo Fisher Scientific). Proteins were denatured at 98 °C for 5 minutes in lithium dodecyl sulfate sample buffer (Thermo Fisher Scientific) and dithiothreitol (Sigma-Aldrich). Western blot was performed using a rabbit anti-GOT2 polyclonal antibody (Bethyl Laboratories, Inc) and a mouse anti-Vinculin monoclonal antibody (Santa Cruz Biotechnology) was used as a loading control.

### ^13^C_6_-Glucose isotope tracing in fibroblasts

Patient and heterozygous fibroblasts were seeded in triplicate in 6-well plates (Corning) and grown for 4 days until ~80-90% confluence. Medium was replaced 24 hours and 72 hours after seeding. On the fourth day, medium was replaced with glucose-free Dulbecco’s Modified Eagle Medium supplemented with 25 mM ^13^C_6_-glucose (Cambridge Isotope Laboratories, Inc) and 0, 2.5, or 5 mM sodium pyruvate (Fluka Chemika, Sigma-Aldrich). After 10 hours, cells were harvested by washing twice with phosphate buffered saline and scraping twice with 250 μL ice-cold methanol. Methanol extracts were collected in 1.5 ml Eppendorf tubes and centrifuged at 16,000 × g at 4 °C for 10 minutes. Supernatants were transferred to fresh Eppendorf tubes and stored at −80 °C.

### Determination of serine and glycine in fibroblasts

For determination of serine, glycine, ^13^C_3_-serine and ^13^C_2_-glycine concentrations in fibroblasts, we adapted the ultra-high performance liquid chromatography tandem mass spectrometry (UPLC-MS/MS) method described by Prinsen et al.^9^ The internal standard mix consisted of ^15^N,^13^C_3_-serine, and ^15^N-^13^C_2_-glycine (Cambridge Isotope laboratories, Inc). Calibration curves were prepared for serine (0-100 μm), glycine (0-500 μm), ^13^C_3_-serine (0-10 μm), and ^13^C_2_-glycine (0-50 μm). In addition, 2 quality controls were prepared from a mixture of cell lysates, either containing high or low concentrations of serine and glycine. 10 μL methanol cell extract was combined with 10 μL internal standard mix and 140 μL solvent A (10 mM ammonium formate in 85% acetonitrile and 0.15% formic acid). Samples were centrifuged for 5 mins at 13,000 rpm and transferred to a 96-well plate (Waters). Samples were measured in technical triplicates. Concentrations were averaged between 2 replicate measurements and corrected for protein concentration determined by a Pierce bicinchoninic acid protein assay (Thermo Fisher Scientific).

### Untargeted metabolomics in dried blood spots

Untargeted metabolomics analysis of dried blood spots was performed using direct infusion high-resolution mass spectrometry according to Haijes et al.^10^ Each sample was measured in duplicate by injecting twice from the same well. Data processing was performed by an in-house metabolomics pipeline in R (Source code available at: https://github.com/UMCUGenetics/DIMS). Mass peaks were annotated by matching the mass over charge ratio (m/z) to metabolite masses in the Human Metabolite Database (HMDB; version 3.6) within a range of 5 parts per million. For each sample, the mean intensity of the duplicate injection was reported for each mass peak. For comparison of features between runs, the intensities were normalized by the sum of all internal standards. Each spot was measured in 2 independent measurements.

### Targeted analysis of amino acids in dried blood spots

Determination of amino acid concentrations in dried blood spots with UPLC-MS/MS was adapted from Prinsen et al.^9^ Of each sample and quality control, a 1.5-mm punch was made and 50 μL of each of the internal standard solutions was added. Samples were ultrasonicated for 20 minutes at room temperature. For calibration standards, 50 μL of each internal standard mix was added to 5 μL calibration standard. Samples and standards were dried under a gentle nitrogen flow at 40 °C and reconstituted in 140 μL solvent A (10 mM ammonium formate in 85% acetonitrile and 0.15% formic acid). A 5 μL sample was injected and final concentrations were corrected with a dilution factor of 3.937 to account for the difference in volume between standards and samples. Each spot was measured in 2 independent measurements.

### Targeted analysis of lactate, pyruvate, and glycerol-3-phosphate in dried blood spots

Concentrations of lactate, pyruvate, and glycerol-3-phosphate in dried blood spots (DBS) were determined using ultrahigh performance high-resolution mass spectrometry (UPLC-HRMS). To this end, a 3-mm punch was made of each spot and 200 μL methanol, as well as 20 μL ^2^H_3_-lactate internal standard (100 μM, Sigma-Aldrich) was added. Samples were ultrasonicated for 20 minutes at room temperature. Calibration standards were prepared in a range from 2 to 100 μM, and a 20 μL internal standard was added. Samples and standards were dried under a gentle nitrogen flow at 40 °C and reconstituted in 25 μL 50/50 acetonitrile/Milli-Q water. Analysis was performed using an Ultimate 3000 UHPLC system (Thermo Fisher Scientific) coupled to a Q Exactive HF hybrid Quadrupole Orbitrap mass spectrometer (Thermo Fisher Scientific) equipped with a heated electrospray ionization source. Chromatographic separation was achieved by injecting 5 μL sample on an Atlantis Premier BEH Z-HILIC column (1.7 μm, 2.1 × 100 mm; Waters). Autosampler temperature was kept at 10 °C and column temperature was kept at 40 °C. Solvent A consisted of 20 mM ammonium bicarbonate (pH 9.00) in Milli-Q water and solvent B consisted of 20 mM ammonium bicarbonate in acetonitrile. The following gradient elution was used: 0 to 5 minutes linear from 90 to 65% B (0.5 ml/min); 5 to 6 minutes isocratic 65% B (0.5 ml/min); 6 to 6.5 minutes linear from 65 to 90% B (0.65 ml/min); 6.5 to 12 minutes isocratic 90% B (0.65 ml/min); 12 to 12.5 minutes isocratic 90% B (0.5 ml/min). The mass spectrometer was operated in positive and negative ionization mode and the following scan parameters were used: resolution of 120,000; automatic gain control target of 1 × 10^6^, maximum injection time of 200 ms, capillary voltage of 4 kV, capillary temperature of 350 °C and S-lens radio frequency level of 75. Data acquisition was performed using Xcalibur software (Thermo Fisher Scientific, Version 3.0). Peak integration was performed using Tracefinder 4.1 software (Thermo Fisher Scientific). Each spot was measured in 2 independent measurements.

### Statistical analysis

Statistical analysis was conducted using Graphpad Prism (Version 10.1.2). Data from dried blood spots and fibroblasts were analyzed using parametric, unpaired, 2-tailed *t* tests. For dried blood spots, patients and controls were grouped for statistical analysis. Data of multiple DBS for 1 individual were averaged before statistical analysis. Data of multiple replicate measurements are reported as mean ± SD. A *P* value of < .05 was considered statistically significant.

## Results

### Clinical characteristics of patients with *GOT2*-related neurodevelopmental disorders

We identified 16 affected individuals (8 females, 8 males) from 10 families with biallelic *GOT2* variants (Figure 1A, Table 1). Of these, 11 are newly identified (F1 to F6), 4 were previously reported with updated follow-up data (F7 to F9), and 1 was previously published without follow-up (F10). The age at last follow-up ranged from 3.5 years to 30 years (median: 10.2 years; IQR: 6). Almost all patients presented with delays in motor and speech development, severe or profound intellectual disability (ID), epilepsy, microcephaly, and hypotonia in infancy that progressed to limb spasticity with axial hypotonia later in life. Early-onset seizures were common, and 13 individuals showed a progressive clinical course, with 13 experiencing a loss of previously acquired milestones.

The prenatal and perinatal histories were largely unremarkable, except for 1 premature infant who required intensive care and ventilation. All 16 infants initially presented with hypotonia, accompanied by global developmental delay and ID in all cases. Neurodevelopmental features were evident, with the entire cohort failing to develop speech.

The majority of patients exhibited severe to profound intellectual disability (81%, 13/16; F1-P1, F5-P2, and F6-P2 had moderate ID), and progressive microcephaly was present in 93% (14/15). Behavioral abnormalities were identified in 88% of affected individuals (14/16), with autism spectrum disorder and irritability being the most common, diagnosed in 56% (9/16), and 44% (7/16) individuals, respectively. Additional behavioral features included hyperactivity, short attention span (2/16), aggression (2/16), self-injurious behaviors (2/16), and stereotypical movements and poor eye contact observed in the absence of a formal autism spectrum disorder diagnosis (1/16).

**Figure 2:**
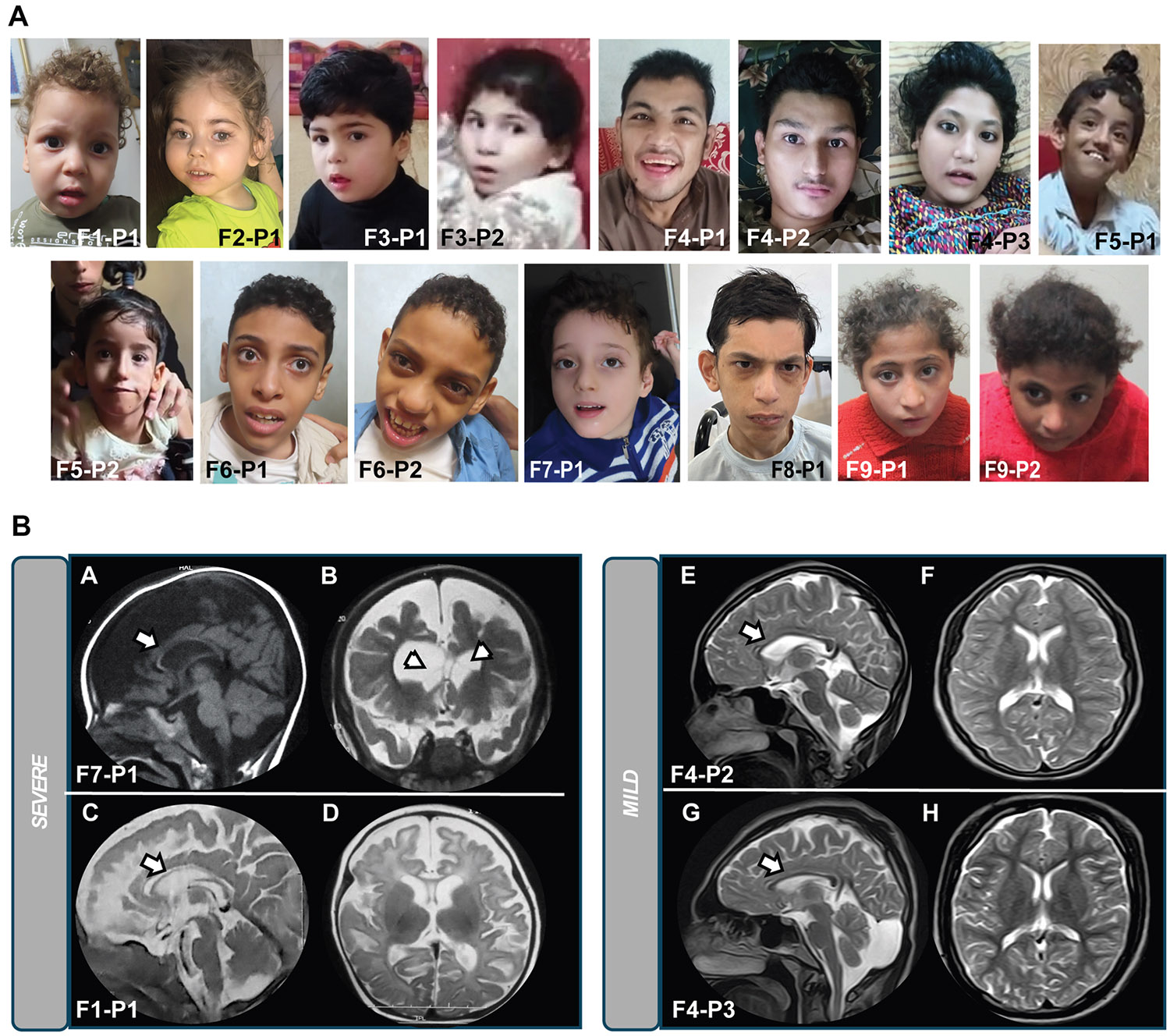
Facial features and brain MRI findings in individuals with *GOT2*-related DEE. A. Facial photographs of 15 affected individuals with *GOT2*-related DEE. B. MRI of 4 different patients divided into severe phenotype (left panel; A-B: #F7-P1; C-D:#F1-P1) and mild phenotype (right panel; E-F:#F4-P2; G-H:#F4-P3). In the left panel, MRI sagittal T1WI (A), coronal T2WI (B), and sagittal and coronal T2WI (C and D) show diffuse cerebral volume loss, mostly in the frontal and parietal lobes, associated with abnormal increase T2WI hyperintensity and ventriculomegaly. Small intraventricular septations are noted (arrowhead, B) and marked thinning of the corpus callosum (arrow, A and C). In the right panel, axial and sagittal T2WI demonstrate mild diffuse cerebral volume loss without abnormal white matter signal change and thinning of the corpus callosum (arrows E and G).

Facial photographs and/or videos from 15 patients across 9 families were systematically reviewed to assess facial dysmorphology. Dysmorphic features were described using the standardized terminology recommended by *Elements of Morphology*, and in cases in which a specific term was unavailable, Human Phenotype Ontology (HPO) terminology was applied. Detailed descriptions of facial dysmorphic features for each patient are presented in Supplemental Table 1, with unique HPO IDs used to tabulate and generate feature frequencies, shown in Supplemental Table 2. Facial photographs of all 15 patients are provided in Figure 2A.

From this analysis, the most common facial dysmorphic features in *GOT2*-related DEE included narrow forehead/bifrontal/bitemporal narrowing (13/15; 86.7%), broad nasal tip (11/15; 73.3%), thin upper lip vermilion (7/15; 46.7%), and tall or pointed chin (10/15; 66.7%). No clearly recognizable facial gestalt was observed in patients with *GOT2*-related DEE, although the patient cohort was relatively small.

Motor delays were universal across the cohort. Of the 16 individuals, 94% (15/16) were nonambulatory, except F4-P1, who began walking at 8 years of age and exhibited an ataxic gait. Spasticity was noted in 94% (15/16), affecting both upper and lower limbs in 83% (5/6) and was often accompanied by axial hypotonia (64%, 9/14). Additionally, 7 individuals (54%, 7/13) demonstrated dystonia, and 50% (7/14) were ataxic. Other features included muscle weakness in 79% (11/14) and muscle atrophy in 85% (11/13). Failure to thrive and short stature were observed in around three-quarters of the cohort (67% and 75%, respectively), including affected siblings from family 10, who exhibited borderline failure to thrive. Feeding difficulties affected 63% (10/16) of patients.

In our cohort, we reviewed neuroimaging from 11 patients, including 7 MRI studies from 5 different families and 4 external MRI reports. Among these, 9 out of 11 patients (81.8%) exhibited varying degrees of cerebral volume loss, and 8 out of 11 (72.7%) showed thinning of the corpus callosum. White matter abnormalities were observed in 7 patients: 4 patients (36.4%) displayed diffuse white matter signal changes involving also the U-fibers, whereas 3 patients (27.3%) had only periventricular white matter signal changes. The signal abnormalities predominantly showed increased signal on T2-weighted imaging with relatively normal T1-weighted signal, indicative of impaired or delayed myelination. Ventriculomegaly was present in 7 patients (63.6%), with 2 cases showing small internal ventricular septations.

The neuroimaging features fell into 2 distinct subgroups (Figure 2B). The first subgroup included patients with severe early-onset presentation, characterized by extensive volume loss, particularly in the frontoparietal regions, thin corpus callosum, and ventriculomegaly with or without septations. The second subgroup presented with milder features, including slight brain volume loss primarily in the periventricular white matter and mild thinning of the corpus callosum. In all patients, the basal ganglia, cerebellum, and brainstem volumes were relatively preserved.

Seizures were nearly universal, with the exception of F4-P1. In 93% of cases (13/14), seizures began within the first year of life (median: 5 months; IQR: 3.5 months). The seizure types varied widely, including tonic, myoclonic, generalized tonic-clonic, clonic, atonic, focal, and absence seizures, with variations seen both between and within individual patients. Seizure control, either partial or complete, was achieved in 87% (13/15) of affected individuals, the majority of whom (14/15) required multiple antiseizure medications (ASMs), except F5-P2, who responded to a single ASM. More than half of the cohort (7/13) received serine and/or pyridoxine (vitamin B6) supplementation as part of their therapeutic regimen. Responsiveness to these supplements, in combination with ASMs, was assessed based on observed changes in seizure frequency and severity following their initiation, typically within complex, multiagent treatment contexts and 86% (6/7) who were on supplementations showed partial or complete seizure control. For example, in F10, seizure frequency markedly declined after the initiation of serine and pyridoxine and ceased after increasing the dose, allowing for complete withdrawal of ASMs by 1.9 years of age. In F7, seizure control was achieved only after introducing both serine and pyridoxine, despite prior use of multiple ASMs and a ketogenic diet. Similarly, F8 demonstrated an approximate 50% reduction in generalized tonic seizures after initiating regular pyridoxine supplementation, without other major treatment changes, suggesting partial responsiveness. On the other hand, F1 had a good seizure control after receiving serine, pyridoxine, ketogenic diet, and ASMs concurrently. In contrast, 2 siblings (F9-P1 and F9-P2) experienced periods of partial seizure control after the addition of pyridoxine, although inconsistent treatment adherence limited further evaluation. In the case of F3, an earlier short trial of pyridoxine during infancy showed no clear benefit. Conversely, 7 affected individuals achieved complete or partial seizure control through ASMs alone, without the use of serine or pyridoxine supplementation (see Supplemental Table 3 for detailed treatment information).

Other common features included bowel and urinary incontinence (100%, 15/15) and sleep disturbances (79%, 11/14). Less common symptoms included joint contractures (F3-P2, F6-P1, F8-P1, F9-P1, and F9-P2), scoliosis (F6-P1, F8-P1, F9-P1, F9-P2, and F10-P1), pica (F4-P1 and F4-P3). Additionally, some individuals experienced gastrointestinal symptoms, such as hematemesis, hiatal hernia with gastroesophageal reflux, and abdominal spasms (F10-P1). Delayed visual evoked potentials and electroretinography responses were noted in F1-P1. For a detailed overview of all clinical characteristics, see Supplemental Table 3.

### Genetic analysis identified segregating *GOT2* biallelic variants in the families

We investigated 15 affected individuals from 9 unrelated families with *GOT2* variants (GenBank: NM_002080.4). Exome sequencing was conducted across all 9 families, including a review of variants from 5 previously reported cases (Figure 1A, Table 2).

Two distinct pathogenic variants were identified across multiple families. In family 1, the proband (F1-IV:2) was found to carry a homozygous pathogenic *GOT2* variant (c.784C>G p.(Arg262Gly)). This same homozygous variant was found in 2 affected brothers in family 6 (F6-IV:1 and F6-IV:2) and 2 affected females in family 9 (F9-III:2 and F9-III:4). The shared Egyptian ethnicity of these 3 families suggests a potential founder mutation. In family 5, a distinct homozygous pathogenic duplication variant encompassing exon 1 of *GOT2* (NC_000016.10: g.58,734,114_58,734,253[4]) was identified in 2 affected females (F5-IV:2 and F5-IV:4).

Three distinct variants were found that were classified as likely pathogenic. In family 3, 2 affected females (F3-III:4 and F3-III:5) were found to carry a homozygous likely pathogenic *GOT2* variant (c.927G>T p.(Lys309Asn)). In family 7, the proband (F7-III:1) was identified with a different homozygous likely pathogenic variant (c.1097G>T p.(Gly366Val)); however, samples of the deceased sister (F7-III:2) were unavailable for testing. In family 8, the affected individual (F8-II:1) was found to carry compound heterozygous likely pathogenic *GOT2* variants (c.[769G>A];[784C>T] p.[(Asp257Asn)]; [(Arg262Cys)])

Two families were found to carry variants of uncertain significance (VUS). In family 2, the affected female (F2-IV:1) carried a homozygous missense variant classified as a VUS (c.538C>T p.(Arg180Trp)). In family 4, the proband (F4-IV:3) was identified with a homozygous VUS (c.1072C>G p.(Leu358Val)) that segregated in 2 additional siblings (F4-IV:3 and F4-IV:5).

To explore a potential link between the positions of the missense and in-frame deletion variants, we mapped them onto both the primary and quaternary protein structures (Figure 1B and C). This analysis revealed a broad distribution of the variants across the primary sequence and 3D protein structure. No distinct patterns or clusters were identified, suggesting the absence of a specific mechanism underlying their pathogenicity. The detailed variant descriptions, zygosity, and segregation patterns are summarized in Table 2, and the pedigrees for all 9 families are illustrated in Figure 1A. Biallelic *GOT2* variants were absent in unaffected family members and either absent or present at very low allele frequencies in several publicly available population databases.^11-14^ Multiple in silico prediction tools supported their deleterious or damaging impact (Table 2).^15-21^ Additionally, these variants were predicted to substitute evolutionarily conserved residues across species (Figure 1D).

We assessed the strength of the gene-disease association for *GOT2* using the ClinGen framework outlined by Strande et al.^22^ We report the presence of several unrelated families harboring biallelic variants in *GOT2* in trans classified as pathogenic or likely pathogenic, of which several have been shown to affect GOT2 expression or function (Supplemental Table 4). These variants are all consistent with an autosomal recessive inheritance pattern and in several instances show moderate evidence of segregation with the disease phenotype. The known function of GOT2 within energy metabolism aligns with the observed clinical features and it has been shown that pathogenic variants in other proteins within the MAS lead to a very similar clinical phenotype. GOT2 expression was shown to be decreased in several patient-derived fibroblast lines, which also showed metabolic alterations consistent with a GOT2 defect. GOT2 knockout and knockdown cell models show a similar metabolic phenotype. Lastly, 2 of the observed homozygous variants were shown to be embryonic lethal in a mouse model. This evidence has been collected in 3 independent studies, including the current one.^6,7^ Taken together, this evidence supports a “Definitive” gene-disease association for *GOT2*.

### Serine and glycine biosynthesis in fibroblasts

**Figure 3:**
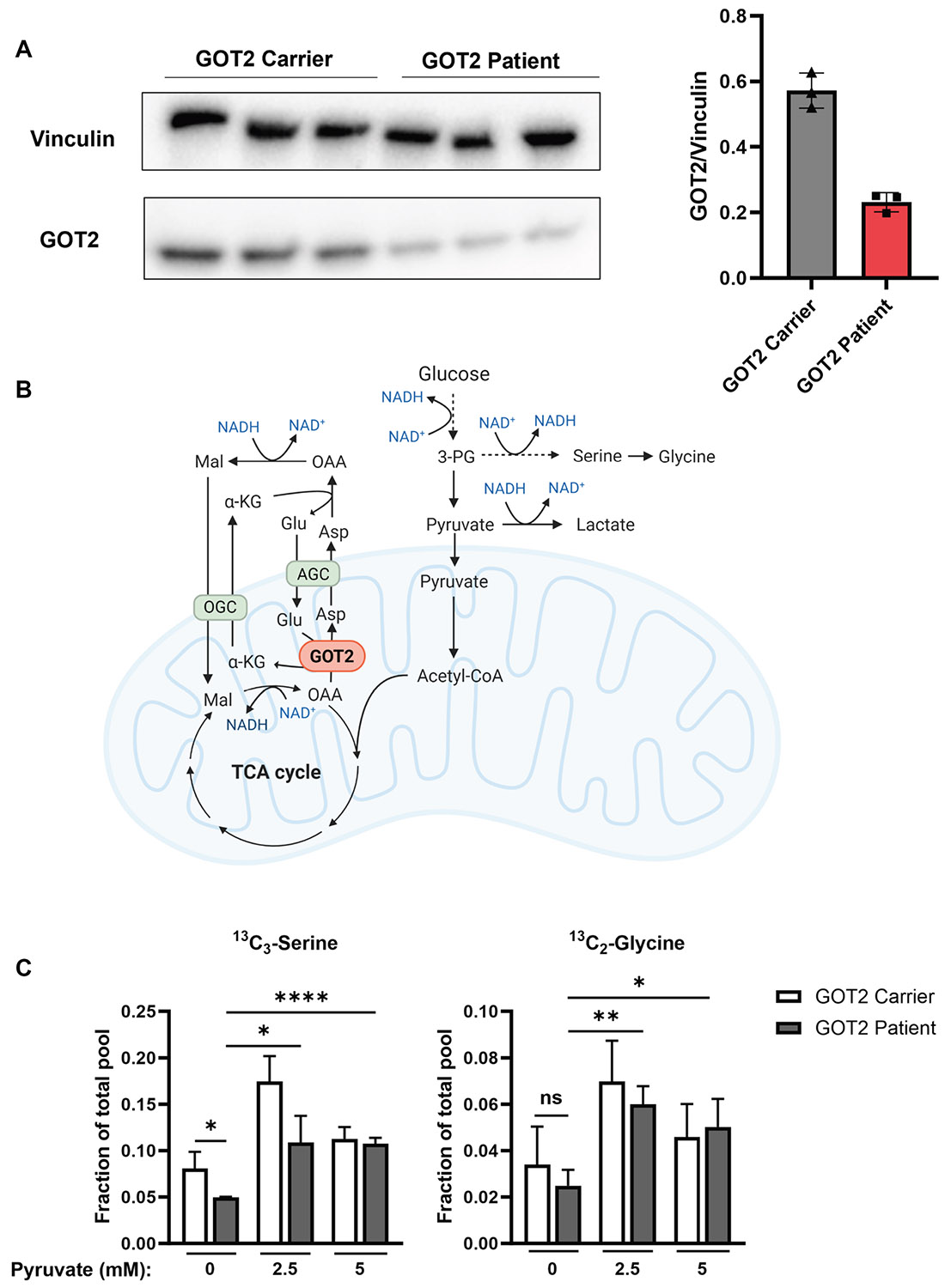
Functional impact of GOT2 deficiency in patient fibroblasts. A. Western blot analysis of GOT2 and loading control Vinculin in fibroblasts from F1-P1 and heterozygote parent. Relative GOT2 expression of patient and heterozygous parent are shown as mean ± SD of technical triplicates. B. Schematic depiction of the role of GOT2 in cellular redox balance. GOT2 transaminates mitochondrial glutamate (Glu) and oxaloacetate (OAA) to α-ketoglutarate (α-KG) and aspartate (Asp), which are transported into the cytosol by the oxoglutarate carrier (OGC) and the aspartate-glutamate carrier (AGC), respectively. In the cytosol, aspartate and α-KG are converted back to OAA and glutamate, after which OAA is reduced to malate (Mal), regenerating cytosolic NAD^+^ . This is used to drive glycolysis, as well as de novo serine biosynthesis. Alternatively, cytosolic NAD^+^ can be regenerated via the conversion of pyruvate to lactate. Cytosolic malate is transported back into the mitochondria by OGC and oxidized to OAA, producing mitochondrial NADH. TCA, tricarboxylic acid; 3-PG, 3-phosphoglycerate; GOT2; aspartate aminotransferase 2. Created using Biorender.com. C. Relative levels of ^13^C_3_-serine and ^13^C_2_-glycine in fibroblasts of F1-P1 and a heterozygote parent after 10 hours of incubation with ^13^C_6_-glucose, with the addition of 0, 2.5, or 5 mM sodium pyruvate. Values are depicted as mean ± SD of technical triplicates averaged from 2 replicate measurements. *P* values were determined by an unpaired, 2-tailed *t* test and are depicted as *P < .05, ^**^ *P* < .01, *** *P* < .001, and **** *P* < .0001.

Previously, it has been shown that serine and glycine biosynthesis in GOT2-deficient fibroblasts and HEK293T GOT2 knockout cells is decreased.^3,6^ To further investigate the extent to which the biosynthesis is affected, we obtained skin fibroblasts from F1-P1 and a heterozygous parent. Samples from other patients in the new cohort were not available for analysis. Using western blot, we confirmed that GOT2 protein levels were decreased in patient fibroblasts, to ca. 40% of heterozygous fibroblasts (Figure 3A).

Serine is synthesized de novo from glycolytic intermediate 3-phosphoglycerate (3-PG; Figure 3B). To investigate the effect of GOT2 deficiency on de novo serine biosynthesis in this patient, the fibroblasts were incubated with ^13^C_6_-glucose and levels of ^13^C_3_-serine and ^13^C_2_-glycine were measured after 10 hours (Figure 3C). The GOT2-deficient fibroblasts indeed showed decreased ^13^C_3_-serine and ^13^C_2_-glycine production, amounting to 62% and 54% of the heterozygous fibroblasts, respectively.

The first step of de novo serine biosynthesis is catalyzed by phosphoglycerate dehydrogenase (PHGDH) in an NAD^+^-dependent manner. Therefore, it has been hypothesized that impaired serine biosynthesis is the result of low cytosolic NAD^+^ availability due to dysfunction of the MAS. Supporting this, it has previously been shown that supplementation of pyruvate to GOT2 knockout HEK293T cells, which can replenish NAD^+^ by conversion to lactate, restores ^13^C_3_-serine production to control levels.^3,6^

Accordingly, when supplemented with 2.5 or 5 mM sodium pyruvate, GOT2-deficient and heterozygous fibroblasts showed significantly increased ^13^C_3_-serine and ^13^C_2_-glycine levels (Figure 3C). This implies that the deficient serine biosynthesis in patient-derived cells is indeed a redox-dependent effect on the activity of PHGDH. Furthermore, the lowest dose of pyruvate is sufficient to fully restore serine biosynthesis to control levels. Additionally, this indicates that NAD^+^-regenerating compounds could form a potentially effective therapy option for GOT2- and other MAS-deficient patients.

### Metabolomics on DBS of patients with GOT2 deficiency

**Figure 4:**
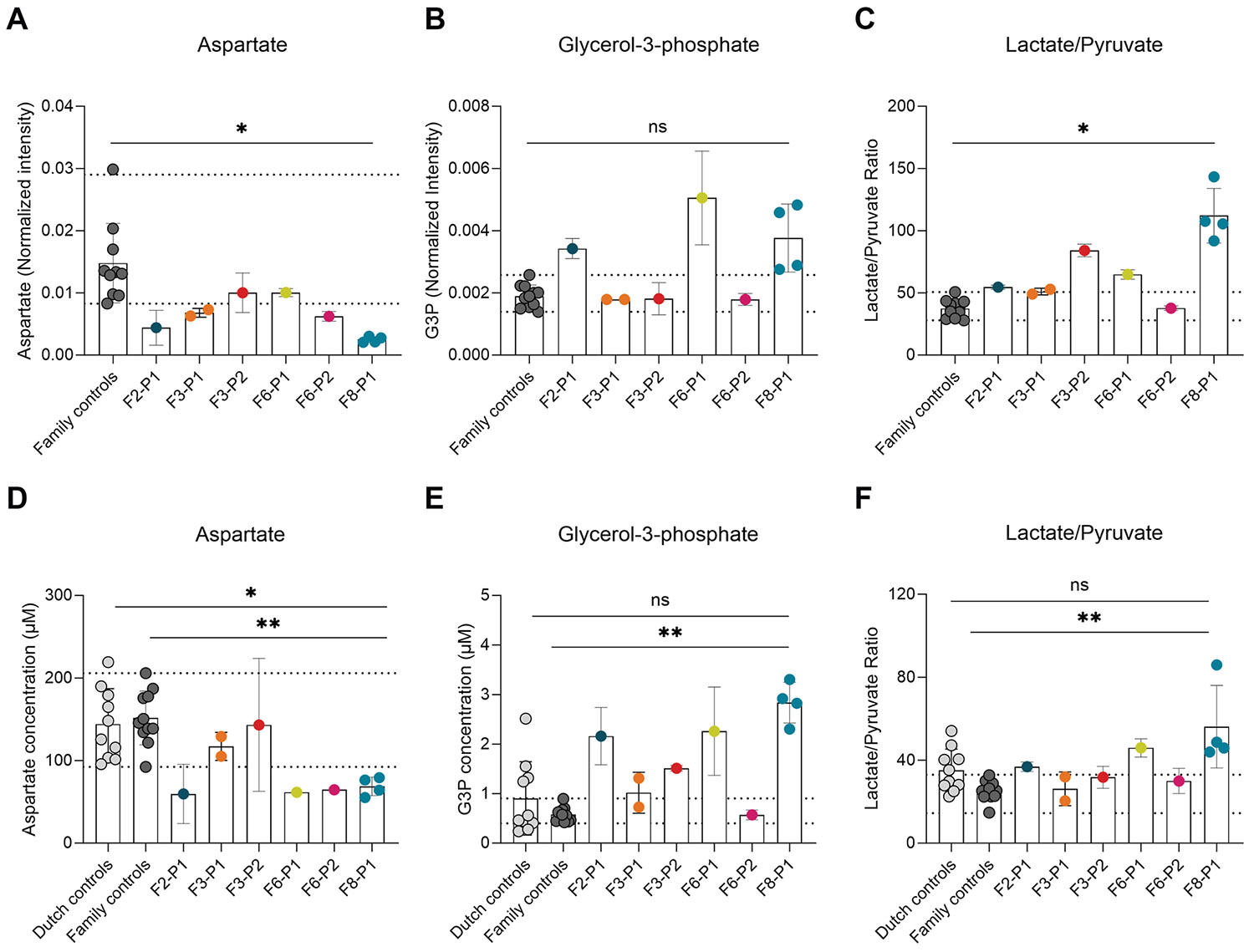
Metabolic markers in dried blood spots of patients with *GOT2*-related DEE. A. Normalized intensities of aspartate and (B) glycerol-3-phosphate and (C) the ratio of lactate and pyruvate in DBS of patients F2-P1 (*n* = 1), F3-P1 (*n* = 2), F3-P2 (*n* = 1), F6-P1 (*n* = 1), F6-P2 (*n* = 1), and F8-P1 (*n* = 4) and family controls (*n* = 11) as determined by direct infusion high-resolution mass spectrometry. Each spot was measured in 2 technical replicates. Dotted lines represent the lower and upper limits of measured family control levels. D. Absolute concentrations of aspartate and (E) glycerol 3-phosphate and (F) the lactate/pyruvate ratio in DBS of patients F2-P1 (*n* = 1), F3-P1 (*n* = 2), F3-P2 (*n* = 1), F6-P1 (*n* = 1), F6-P2 (*n* = 1), and F8-P1 (*n* = 4); family controls (*n* = 11); and Dutch controls (*n* = 10) as determined by UPLC-(HR)MS. Each spot was measured in 2 technical replicates. Dotted lines represent the lower and upper limits of measured family control levels. G. Normalized intensity of serine in patients and family controls as determined by direct infusion high-resolution mass spectrometry and (H) concentration of serine in DBS of Dutch controls, family controls, and patients as determined by UPLC-MS/MS. Each spot was measured in 2 technical replicates. Dotted lines represent the lower and upper limits of measured family control levels. *P* values were determined by performing an unpaired *t* test of the controls versus the group of patients. *P* values are indicated as * *P* < .05, ** *P* < .01, *** *P* < .001, and **** *P* < .0001.

To look further into the metabolic consequences of GOT2 deficiency and find potential novel biomarkers, we analyzed dried blood spots of 6 patients (F2-P1, F3-P1, F3-P2, F6-P1, F6-P2, and F8-P1) and 7 unaffected family members using untargeted direct infusion high-resolution mass spectrometry. One of the most significantly decreased metabolites within the patient group was aspartate, one of the products of GOT2 (Figure 4A). Aspartate was decreased in 4 out of the 6 patients (F2-P1, F3-P1, F6-P2, and F8-P1) compared with the family controls. Targeted UPLC-MS/MS analysis confirmed low aspartate in 3 patients (F2-P1, F6-P2, and F8-P1), and showed decreased aspartate for F6-P1 as well. Aspartate concentrations for F3-P1 and F3-P2 were within the range of the family and Dutch controls (Figure 4D). Untargeted analysis also revealed high glycerol-3-phosphate (G3P) in 3 patients (Figure 4B; F2-P1, F6-P1, and F8-P1), as well as an increased lactate/pyruvate ratio for F2-P1, F3-P2, F6-P1, and F8-P1 (Figure 4C). G3P is formed from glycolytic intermediate dihydroxyacetone phosphate by the enzyme glycerol-phosphate dehydrogenase in an NADH-dependent reaction. Therefore, similarly to lactate, accumulation of G3P could be indicative of a decreased cytosolic NAD^+^/NADH ratio. Accordingly, it has previously been shown that GOT2 knockout HEK293T cells have increased ^13^C_3_-G3P and ^13^C_3_-lactate production from ^13^C_6_-glucose.^3^ To confirm these findings in a targeted setting, UPLC-HRMS was used to determine pyruvate, lactate and G3P concentrations (Figure 4E and F). This confirmed the increased lactate/pyruvate ratio in 3 patients (F2-P1, F6-P1, and F8-P1), as well as increased G3P levels in 4 patients (F2-P1, F3-P2, F6-P1, and F8-P1). For F3-P1, G3P concentrations and the lactate/pyruvate ratio were elevated in 1 of 2 DBS, whereas they were normal in the other. Overall, the patient group showed significantly decreased levels of aspartate and significantly increased levels of G3P compared with healthy family members (*P* < .05). For several patients, however, considerable overlap was observed with the control group, suggesting limited value of these metabolites as standalone diagnostic markers.

Lastly, we investigated serine levels in dried blood spots. Surprisingly, untargeted metabolomics showed unaltered serine levels in most patients (Figure 4G). F6-P1 had increased levels compared with controls. Targeted analysis showed serine concentrations within the normal range for F2-P1, F3-P2, and F6-P2 and increased concentrations for F3-P1, F6-P1 and 3 out 4 spots of F8-P1 (Figure 4H).

## Discussion

Inborn errors of the MAS have emerged in recent years as a cause of severe early-onset epileptic encephalopathy, brain MRI abnormalities, developmental delay, and lactic acidosis. These include deficiency of malate dehydrogenase 1 (*MDH1*; OMIM 618959)^23^ and 2 (*MDH2*; OMIM 617339),^24-27^ AGC1/Aralar (OMIM 612949),^28-37^ and GOT2 (OMIM 618721).^6,7^ For an overview of these disorders including known variants, biochemical markers and neuroimaging findings, see Supplemental Table 5. Deficiency of Citrin/AGC2 (OMIM 603859), the liver isoform of aspartate-glutamate carrier (AGC), leads to a hepatic clinical phenotype.^38^ Deficiency of GOT1 (OMIM 138180) has not been described, but there are known variants associated with early-onset severe preeclampsia,^39^ elevation of serum aspartate aminotransferase levels (macro-AST),^40^ and decreased serum AST activity.^41^

This study provides a comprehensive clinical review of newly identified individuals with *GOT2*-related disorder, along with additional and follow-up data from previously published cases, encompassing a total of 16 individuals. Our findings confirm and expand upon prior reports, defining a core phenotype that includes global developmental delay/ID (GDD/ID), seizures, progressive microcephaly, infantile hypotonia evolving into limb spasticity and axial hypotonia, minimal or absent speech, behavioral abnormalities, and dysmorphic facial features.

Although GDD/ID was a universal feature, the severity varied across individuals, indicating greater clinical heterogeneity than previously appreciated. This variability may not have been evident in earlier reports because of smaller cohort sizes or limited longitudinal follow-up. Motor impairments were consistently observed, and although independent ambulation was largely absent, rare instances of delayed walking suggest some variability in motor outcomes, as reported in other MAS-related conditions.^42,43^

Neurological findings demonstrated evolution of tone abnormalities, with most individuals progressing from early hypotonia to limb spasticity and axial hypotonia. However, the presentation was not uniform, and some individuals retained hypotonia without developing spasticity, suggesting either phenotypic expansion or early-stage assessments. Interestingly, follow-up data, with the addition of new cases, revealed the presence of movement disorders, such as dystonia and ataxia, in a significant proportion of individuals, further expanding the phenotypic spectrum. These findings might also suggest shared pathophysiological mechanisms across MAS-related disorders, including AGC1 deficiency, for which similar tonus evolution is documented.^42,43^ Epilepsy emerged as a prominent and often early feature, although clinical presentations and treatment responses varied. Although treatment outcomes varied, supplementation with serine and pyridoxine appeared beneficial in most of the cases. Furthermore, 2 patients who were not on these supplements demonstrated poor seizure control despite multiple anti-epileptic medications. On the other hand, seizure control was achieved through nonsupplement ASMs alone in some cases. Overall, these findings might suggest a complex interplay between metabolic dysfunction and seizure pathophysiology, for which intervention may be critical but not solely determinative of therapeutic success and further support the need of a multifaceted approach to seizure management in *GOT2*-related disorder. Nonneurological manifestations were also prominent, including failure to thrive, short stature, feeding difficulties (such as swallowing issues and intolerance), and behavioral abnormalities, further complicating care, and highlighting the need for comprehensive management strategies that address neurological, nutritional, and behavioral needs.

Facial dysmorphology in GOT2 deficiency appears distinctive within MAS-related disorders. Common features included microcephaly, narrow forehead, broad nasal tip, thin upper lip, and a tall or pointed chin. In comparison, MDH2 deficiency shows no documented dysmorphic features. MDH1 deficiency, reported in 2 siblings, shares overlapping traits, such as microcephaly, broad nasal tip, and frontal bossing. AGC1 deficiency presents limited and inconsistent dysmorphic data.

Our findings suggest that *GOT2*-related dysmorphology may have unique characteristics, warranting further studies with larger cohorts to establish a clearer phenotypic spectrum across MAS deficiencies.

Through neuroimaging analysis, we could distinguish 2 groups based on disease severity. This included severe forms in which extensive brain degenerative changes were observed in the first years of life, characterized mostly by diffuse cerebral volume loss—particularly in the frontal and parietal lobes—along with periventricular and subcortical white matter signal changes with marked thinning of the corpus callosum and the milder forms presented in older children with minimal associated cerebral volume loss without significant white matter signal changes. Notably, when present, white matter signal abnormalities were primarily noted on T2WI, with relative normal appearance on T1WI. These findings indicate an impairment in the normal myelination process rather than an acute encephaloclastic injury and are suggestive of hypomyelination. However, it is worth to mention that in this scenario that is often associated cortical atrophy, these white matter abnormalities are most likely related to the superimposed neuronal involvement in the disease instead of a primary hypomyelination process, which follows a similar discussion for neuroimaging features observed in patients with *AGC1* pathogenic variants.^32^

Our findings are in line with the current neuroimaging literature description of 5 patients with disease-related *GOT2* pathogenic variants,^6,7^ except for 1 patient described presenting extensive areas of cystic encephalomalacia along with changes in the basal ganglia and thalamus, features not observed in our cohort.

Interestingly, our findings are also very similar to those observed in patients related to other disorders affecting the MAS pathway, such as *MDH1*, *MDH2*, and *AGC1* (*SLC25A12*), including the presence of enlargement of the lateral ventricles—predominantly along the frontal horns—thinning of the corpus callosum, white matter volume loss with features indicating impairment of the myelination, and ventricular septations, which gives further support for the role of the same disease process across those different genes.

Although some degree of atrophy was a consistent feature across all of our patients, the basal ganglia, thalami, brainstem, and cerebellum exhibited relatively normal signal intensity and volume, regardless of the severity of the presentation. These were also frequent findings for other pathogenic variants related to MAS function, except for 1 *MDH2*-related patient described with selective cerebellar atrophy^25^ and 1 *AGC1*-related patient with unremarkable findings^33^ (Supplemental Table 5).

Moreover, similar to patients with MAS deficiency, individuals presenting with the neuropathic form of pyruvate dehydrogenase complex deficiency also tend to present early with severe neurological impairment and lactic acidosis, sharing similar imaging findings such as diffuse white matter volume loss, ventriculomegaly, and intraventricular septations. However, unlike GOT2, MDH1, MDH2, and AGC1, patients with pyruvate dehydrogenase complex deficiency often present with additional malformative commissural features, including dysgenesis or agenesis of the corpus callosum and cavitations in the ganglionic eminences,^44^ findings not observed in our cohort.

A few additional differentials should be considered from the neuroimaging perspective. The literature description of 1 patient with multicystic encephalomalacia and a few additional patients exhibiting signal abnormalities in the bilateral posterior putamina and subtle abnormal signals in both thalami allow us to consider *GOT2*-related disorder as a potential mimicker of neonates with hypoxic-ischemic injury and other classic hypoxic-ischemic injury mimickers, including sulfite oxidase deficiency and molybdenum cofactor deficiency.^45^

Interestingly, all currently documented cases of GOT2 deficiency involve biallelic missense variants or in-frame deletions, which are predicted, and in some cases confirmed, to result in partial loss of GOT2 protein levels and enzymatic function. To date, no biallelic variants in *GOT2* have been identified that result in a complete loss-of-protein function. This is consistent with findings for other MAS enzyme deficiencies, for which similar complete loss-of-function variants have not been observed. This can likely be attributed to the fact that complete loss of function of GOT2, or any of the other 5 MAS enzymes, is lethal. This is in line with the crucial role of the MAS in maintaining cellular energy metabolism and redox homeostasis.

Biochemically, GOT2 deficiency led to a secondary serine biosynthesis defect in previously reported patients, which we also found in fibroblasts of F1-P1. The fact that this was only conducted in fibroblasts from a single patient from the new cohort is however a limitation. Therefore, future work should aim to include a broader set of patient-derived fibroblasts to strengthen and further validate these observations. Despite this, a recent study showed that dysfunction of the MAS in HEK293T decreases serine biosynthesis through a decreased NAD^+^/NADH ratio.^3^ This, in turn, hinders the reaction of PHGDH, the NAD^+^-dependent rate-limiting enzyme of de novo serine and glycine biosynthesis. Addition of pyruvate to MDH1- and 2 and GOT2-deficient HEK293T cells resulted in normalization of serine biosynthesis. This finding is supported by the results obtained in our study, in which pyruvate supplementation increased serine and glycine biosynthesis in patient-derived cells. This shows that increasing cytosolic NAD^+^ levels can boost serine production through PHGDH in a patient background. Furthermore, pyruvate could contribute to aspartate production via the subsequent reactions of pyruvate carboxylase and GOT1. Because pyruvate supplementation may lead to excessive lactate production, other NAD^+^-regenerating compounds could be considered. Van Karnebeek et al^6^ suggest the use of medium-chain triglycerides as an alternative source of energy because oxidation of these fatty acids only produces NADH in the mitochondria and therefore does not require cytosolic NADH oxidation. Triheptanoin, the triglyceride of heptanoic acid (C7), has already shown positive results in 1 patient with MDH2 deficiency.^27^ Further research should be conducted to assess whether it would also be beneficial for patients with GOT2 deficiency.

Serine is an important precursor for nucleotide biosynthesis through the production of folates and for other amino acids, including glycine, cysteine, and tryptophan. Furthermore, it has an essential function as a neurotransmitter in the brain, which may partially explain the seizure phenotype. Accordingly, inborn deficiencies of enzymes in de novo serine biosynthesis clinically resemble GOT2 deficiency, with the most important symptoms being encephalopathy, epilepsy, and hypotonia.

Besides disturbed serine biosynthesis in fibroblasts, high lactate/pyruvate ratio and high glycerol-3-phosphate levels found in DBS of several patients are also indicative of a disturbed NAD^+^/NADH ratio. Because both pathways are NADH-dependent, the elevation of their products can be ascribed to the impaired recycling of cytosolic NAD^+^ by the MAS. High lactate/pyruvate ratio has been reported in MDH1 and −2 deficiency, as well as previously described GOT2 deficient patients.^6,23,24^ Elevated G3P, however, has not been described before for GOT2 deficiency and could thus be a valuable novel biomarker because it was found in all patients analyzed in this new cohort. High G3P levels have been reported for MDH1 deficient patients, as well as in knockout cells of all individual MAS components, indicating that it is an important hallmark of MAS dysfunction.^3,23^

Low aspartate levels were found in DBS of 4 out of 6 patients. This has not been reported previously in patients, potentially because amino acids are not routinely measured in DBS. Decreased aspartate has been shown in *GOT2* knockdown and knockout cell models and could be a valuable marker pointing toward a defect in GOT2.^3,46^ Low aspartate could have several consequences, the main one being disruption of the urea cycle. Mitochondrial aspartate is transported into the cytosol by AGC, after which it is combined with citrulline by argininosuccinate synthetase to form argininosuccinate. When aspartate is low, this can lead to hypercitrullinemia and consequently hyperammonemia due to dysfunction of the urea cycle. These symptoms have been reported in patients who previously received a diagnosis of GOT2 deficiency and in patients with AGC deficiency.^6,38^ Because cells generally do not take up high amounts of aspartate from the blood, they are highly dependent on aspartate production. Therefore, aspartate supplementation may not be a suitable treatment option.^47^

Aspartate also functions as an excitatory neurotransmitter in the brain, as well as a precursor for N-acetyl-aspartic acid (NAA), of which the role is still poorly understood. In patients deficient in AGC1, the observed hypomyelination was initially linked to low levels of cytosolic aspartate and NAA.^48^ However, administration of β-hydroxybutyrate to an Aralar KO mouse model improved myelination without increasing brain aspartate or NAA levels^49^; therefore, a link remains unlikely.

Although levels of aspartate and G3P showed statistically significant differences between patient and control groups, the observed overlap with healthy individuals suggests that they may not be suitable as independent screening biomarkers. Rather, they may serve as supportive metabolic indicators that are interpreted alongside clinical and genetic findings.

Surprisingly, serine levels in DBS were found to be normal or even elevated, despite the apparent defect in de novo biosynthesis. This can potentially be attributed to serine treatment or dietary serine intake, both of which can normalize blood serine levels. Consequently, assessing levels of serine in cerebrospinal fluid may provide more meaningful insights because these are less influenced by dietary intake and reflect the important role of serine in the brain.

Timely diagnosis of MAS deficiencies remains challenging because the patient groups are small and specific clinical and biochemical symptoms are still lacking. They are characterized by altered levels of a wide range of metabolites in different pathways because the MAS has such a wide-spread effect on the metabolic status of a cell because of its important role in redox homeostasis. Therefore, it is important to keep expanding our knowledge on the phenotypic and genotypic spectrum of MAS disorders and continue the search for novel biomarkers and treatment options.

This study significantly expands the clinical and molecular understanding of *GOT2*-related disorders, emphasizing its severe yet variable neurodevelopmental and neurodegenerative phenotype and highlighting its similarities with other disorders of the MAS. The findings underscore the importance of early diagnosis and targeted therapies, particularly with serine and pyridoxine supplementation. Comparative analysis with other MAS-related disorders provides valuable insights into shared mechanisms and clinical features. Continued international collaboration and comprehensive follow-up are crucial to further delineate the phenotype, enhance patient care, and advance research into therapeutic approaches.

## Supplementary Material

Supp Table S1

Supp Table S2

Supp Table S3

Supp Table S4

Supp Table S5

## Figures and Tables

**Table 1:** Summary of clinical characteristics of all 14 affected individuals

Affected Individual (Family-Patient)	F1-P1	F2-P1	F3- P1	F3- P2	F4-P1	F4-P2	F4-P3	F5-P1	F5- P2	F6-P1	F6- P2	F7-P1^a^	F8-P1^b^	F9- P1^c^	P2^d^	F10-P1^e^	%
Variant^h^	p.Arg262Gly	p.Arg180Trp	p.Lys309Asn			p.Leu358Val		dup of exon 1		p.Arg262Gly		p.Gly366Val	p.Asp257Asn/p.Arg262Cys	p.Arg262Gly		p.Leu209del/p.Arg337Gly	X
Gender	M	F	F	F	M	M	F	F	F	M	M	M	M	F	F	M	50% F 50% M
Age	6y	3y9m	5y4m	3y6m	28y	19y	14y	11y2m	9y5m	12y	7y8m	8y	30y	13y	11y	8y	Range: 3y6m-30y
GDD/ID (severity)	2+	4+	4+	4+	3+	3+	3+	3+	2+	3+	2+	3+	4+	3+	3+	4+	100%
Prog. microcephaly	+	+	−	X	+	+	+	+	+	+	+	+	+	+	+	+	93%
Behav. abn.	+	+	+	−	+	+	−	+	+	+	+	+	+	+	+	+	88%
Prog. course	+	+	+	X	+	+	+	+	+	+	+	X	+	+	+	X	100%
Hypotonia in	+	+	+	+	+	+	+	+	+	+	+	+	+	+	+	+	100%
infancy																	
Spasticity	+ (LL)	−	+	+	+ (UL, LL)	+ (UL, LL)	+ (UL, LL)	+	+	+	+	+	+ (UL, LL)	+	+	+ (UL, LL)	94%
Axial hypotonia	−	+	X	X	+	+	+	+	+	−	−	+	+	−	−	+	64%
Independent	−	−	−	−	+	−	−	−	−	−	−	−	−	−	−	−	6%
ambulation																	
Seizures/age of	+/2	+/4	+/6	+/6	−	+/24	+/X	+/3	+/4	+/4	+/7	+/3	+/3	+/7	+/6	+/9	94%
onset (months)																	
Treatment response	+	−	−	+	X	+	+	+	P	P	P	+	P	+	+	+	87%^f^
Serine supp.	+	−	−	−	X	−	−	−	−	X	X	+	X	−	−	+	25%
Pyridoxine supp.	+	−	+	−	X	−	−	−	−	X	X	+	+	+	+	+	54%
Ataxia	−	−	+	−	+	+	+	+	+	−	−	+	X	−	−	X	50%
Dystonia	−	−	X	X	−	−	−	+	+	+	+	+	−	+	+	X	54%
FTT/short stature	−/+	+/−	−/−	X/−	−/+	−/−	−/+	+/+	+/+	* /+	* /+	+/+	+/+	+/+	+/+	+/+	67%^g^/75%
Dysmorphism	+	+	+	+	+	+	+	+	+	+	+	+	+	+	+	X	100%
Brain abn.	+	+	+	−	X	+	+	+	+	+	+	+	+	+	+	+	93%

* , borderline; *behav. abn*., behavioral abnormalities; *brain abn*., brain abnormalities; *F*, female; *FTT*, failure to thrive; *GDD*, global developmental delay; *ID*, intellectual disability; *LL*, lower limb; *m*, months *M*, male; *P*, partial; *prog*., progressive; *supp*., supplementation; *UL*, upper limb; *X*, not available or not applicable; *y*, years. For the severity: +, mild; 2+, moderate; 3+, severe; and 4+, profound.

a Follow-up data for Individual 4, family III from van Karnebeek et al,^6^ 2019.

b Follow-up data for Çapan et al,^7^ 2023.

c Follow-up data for individual 2, family II from van Karnebeek et al,^6^ 2019.

d Follow-up data for individual 3, family II from van Karnebeek et al,^6^ 2019.

e Individual 1, family I from van Karnebeek et al,^6^ 2019.

f Includes both response to treatment and partial response.

g Includes borderline cases.

h Detailed annotations in Table 2.

**Table 2:** Table of *GOT2* variants

Characteristics	Family 1	Family 2	Family 3	Family 4	Family 5	Family 6	Family 7	Family 8		Family 9
Ethnicity	Egyptian	Iranian	Syrian	Pakistani	Yemeni	Egyptian	Egyptian	Turkish		Egyptian
Method of identification	Exome sequencing	Exome sequencing	Exome sequencing	Proband only exome sequencing	Exome sequencing	Exome sequencing	Probands exome sequencing	Proband only exome sequencing		Probands exome sequencing
Method of validation	Sanger sequencing	Sanger sequencing	Sanger sequencing	Sanger sequencing	Sanger sequencing	Sanger sequencing	Probands exome sequencing	Sanger sequencing		probands exome sequencing
Genomic position (GRCh38/hg38- NC_000016.10)	g.58750636G>C	g.58752490G>A	g.58750010C>A	g.58743419G>C	g.58734114_58734253 [4]	g.58750636G>C	g.58743394C>A	g.58750651C>T (pat)	g.58750636G>A (mat)	g.58750636G>C
Coding sequence change (NM_002080.4)	c.784C>G	c.538C>T	c.927G>T	c.1072C>G	NA	c.784C>G	c.1097G>T	c.769G>A	c.784C>T	c.784C>G
Protein sequence change (ENSP00000217446.3)	p.(Arg262Gly)	p.(Arg180Trp)	p.(Lys309Asn)	p.(Leu358Val)	NA	p.(Arg262Gly)	p.(Gly366Val)	p.(Asp257Asn)	p.(Arg262Cys)	p.(Arg262Gly)
Exon number position	7 of 10	5 of 10	8 of 10	9 of 10	1 of 10	7 of 10	9 of 10	7 of 10	7 of 10	7 of 10
Codon change	Cgc/Ggc	Cgg/Tgg	aaG/aa	Ctg/Gtg	139 bp-ins[4]	Cgc/Ggc	gGt/gTt	Gat/Aat	Cgc/Tgc	Cgc/Ggc
Consequence	Missense	Missense	Missense	Missense	Duplication	Missense	Missense	Missense		Missense
Zygosity	Homozygous	Homozygous	Homozygous	Homozygous	Homozygous	Homozygous	Homozygous	Compound heterozygous		Homozygous
gnomAD v4.1	0	1.31 e-6	0	1.37 e-6	0	0	6.84 e-7	0	2.05 e-6 [3,0]	0
gnomAD v3	0	0	0	0	0	0	0	0	0	0
BRAVO TOPMed Freeze 8	0	7.56 e-6 [2,0]	0	0	0	0	0	0	7.56 e-6 [2,0]	0
100,000 GPRD	0		0	0	0	0	0	0	0	0
jMorp	0		0	0	0	0	0	0	0	0
UK Biobank	0		0	0	0	0	0	0	0	0
Total allele (het+homo)	0	3	0	2	0	0	1	0	0	0
GERP	5.54	5.73	3.3	3.32	NA	5.54	5.25	5.54	5.54	5.54
CADD_Phred	29.5	32	26.7	24.4	NA	29.5	28	30	32	29.5
Polyphen-2	PD (0.9058)	PD (0.9058)	PD (0.7322)	PD(0.4661	NA	PD (0.9058)	PD (0.6557)	PD (0.9058)	PD (0.9058)	PD (0.9058)
SIFT	PS (0)	PS (0)	PS (0.001)	PS (0)	NA	PS (0)	PS (0)	PS (0)	PS (0)	PS (0)
PROVEAN	PM (−6.94)	PM (−7.36)	PS (−4.8)	U(−2.92)	NA	PM (−6.94)	PM (−8.6)	PS (−4.95)	PM (−7.91)	PM (−6.94)
MutationTaster	DC (0.8100)	DC (0.8100)	DC (0.8100)	DC(0.8100)	NA	DC (0.8100)	DC (0.8100)	U (1)	DC (0.8100)	DC (0.8100)
ACMG/AMP criteria^a^	PM2, PP3 (S), PP5 (VS)	PM2, PP3 (M)	PM1. PM2, PP3 (M)	PM2, PP3 (Moderate)	PP4	PM2, PP3 (S), PP5 (VS)	PM2, PP3 (M), PP5 (VS)	PM2, PP3 (S)	PM2, BP4 (S)	PM2, PP3 (S), PP5 (VS)
ACMG/AMP verdict	P	VUS	LP	LP	P	P	P	LP	LP	P

Detailed molecular characteristics of identified variants.

a Moderate: M, Strong: S, and Very Strong: VS.

## Data Availability

The authors confirm that the data supporting the findings of this study are available within the manuscript and its supplemental data.
