# Supplementary material for: Comprehensive genotypic, phenotypic, and biochemical characterization of GOT2 deficiency: A progressive neurodevelopmental disorder with epilepsy and abnormal movements
Source: Genet Med. Author manuscript; Available in PMC 2026 Apr 24. (PMC13108455; doi:10.1016/j.gim.2025.101587)
Supplement: Supp Table S4 [file NIHMS2163352-supplement-Supp_Table_S4.pdf]

Supplementary Material

Supplementary Table 4.

Assertion Criteria	Genetic Evidence (0-12 Points)	Points	Experimental Evidence (0-6 Points)	Points	Replication over time	References
**Description**	Case-level, segregation or case-control data supporting gene-disease association		Gene-level experimental evidence supporting gene-disease association		>2 publications with convincing evidence over time (>3 yrs) by independent research groups	
**Evidence**	Several families with two variants in trans with at least one predicted null variant	5	Protein function and expression is consistent with disease phenotype	1	YES<br>(6), (7), this study	
	Limited evidence of variant segregation with disease phenotype	6	Variants in related proteins cause similar disease phenotype	0.5		(22)-(36)
			Expression and function of protein are altered in patient-derived cells; rescued in one patient	1		(6), this study
			Animal and cell-culture model with disruption of protein show a similar phenotype	4		(3), (6)
**Assigned points**		11		6		
**Total Points**	**17**	**Calculated Classification**	**DEFINITIVE (12-18 pts)**			
